# Association of Physician Characteristics With Perceptions and Experiences of Gender Equity in an Academic Internal Medicine Department

**DOI:** 10.1001/jamanetworkopen.2019.15165

**Published:** 2019-11-13

**Authors:** Shannon M. Ruzycki, Georgina Freeman, Aleem Bharwani, Allison Brown

**Affiliations:** 1 Division of General Internal Medicine, Department of Medicine, University of Calgary, Calgary, Alberta, Canada; 2 Department of Community Health Sciences, University of Calgary, Calgary, Alberta, Canada; 3 W21C Research and Innovation Centre, O’Brien Institute for Public Health, University of Calgary, Calgary, Alberta, Canada

## Abstract

**Importance:**

The persistence of inequities that disadvantage women physicians remains empirically underexplained. Understanding the cultural factors that are associated with disparities in harassment, discrimination, remuneration, and career trajectory are critical to addressing inequities.

**Objectives:**

To explore how physicians perceive the climate for women physicians and compare perceptions and experiences of gender inequity among physicians based on characteristics including gender, faculty status, parental status, and years in practice.

**Design, Setting, and Participants:**

This sequential, explanatory, mixed-methods qualitative study used the Culture Conducive to Women’s Academic Success (CCWAS; range 45-225, with higher scores indicating better perceived culture toward women), followed by individual semistructured interviews with physicians at the Department of Medicine of the University of Calgary. All 389 physician members of the Department of Medicine, including academic and clinical physicians and those of any gender, were invited to participate in the survey and interview phases.

**Main Outcomes and Measures:**

The culture within the department for women physicians was assessed using the CCWAS score. Scores were compared between respondents’ gender and years in practice. Interviews with physicians were used to further explore findings from the CCWAS and to understand experiences and perceptions of gender disparities.

**Results:**

A total of 169 of 389 physicians completed the survey (response rate, 43.4%; 102 [59.9%] women; 65 [38.9%] men; and 2 [1.2%] who did not disclose gender); 28 participants (7.2%) elected to participate in an interview (22 [78.6%] women; 6 [21.4%] men). Women physicians perceived the culture of the department toward women as significantly worse than men physicians (median [interquartile range] CCWAS score, 137.0 [118.0-155.0] vs 164.5 [154.0-183.4]; *P* < .001). Physicians with more than 15 years in practice perceived the culture toward women as significantly more favorable than physicians with 15 years or less in practice (median [interquartile range] CCWAS score, 157.0 [138.8-181.3] vs 147.0 [127.5-164.3]; *P* = .02). Qualitative data demonstrated that experiences of junior women (ie, physicians who graduated medical school after 1996, when an equal number of men and women in medical school was achieved in Canada) and perceptions of senior men (ie, those who graduated before 1996) were most different; junior women reported high rates of discrimination and harassment, while senior men perceived that the Department of Medicine had achieved gender equity.

**Conclusions and Relevance:**

In this study, senior men physicians’ perceptions of gender equity were different from lived experiences of gender inequity reported by junior women physicians. This demographic mismatch between perceptions and experiences of gender equity in medicine may explain the lack of action by leaders and decision-makers in medicine to mitigate disparities.

## Key Points

**Question:**

Are there gender-based and generational differences in how physicians in the Department of Medicine at the University of Calgary perceive and experience gender inequity?

**Findings:**

This mixed-methods study demonstrated that men physicians perceive that the culture is more favorable for women than women physicians. Junior women physicians reported experiencing greater bias and discrimination compared with their men peers, while senior men physicians perceived little or no gender inequity for women physicians.

**Meaning:**

This study suggests that there are gender-based and generational differences in how gender inequities are perceived and experienced in medicine.

## Teaser

This mixed-methods qualitative study explores how physicians in the Department of Medicine at the University of Calgary perceive the climate for women physicians and compares perceptions and experience of gender inequity among physicians based on characteristics including gender, faculty status, parental status, and years in practice.

## Introduction

Gender disparities that disadvantage women physicians in compensation, leadership attainment, and experiences of discrimination are well described.^1,2,3,4,5^ Compelling evidence of a gender gap in remuneration, academic promotion, and harassment has existed since at least 1990.^6,7,8^ Despite 23 years of numerical equality between women and men in medical school, contemporary Canadian data demonstrate gender disparities in pay, leadership, and harassment for women physicians and academics.^9,10,11,12,13,14,15,16,17,18,19^ These data suggest that additional factors, beyond the number of female physicians available to enter leadership tracks and the awareness of the gender gap, contribute to ongoing inequities.

Several explanations have been proposed for persistent disparities, including systemic harassment and discrimination against women physicians, misalignment of women physician’s personal values with the objectives of leadership, and work-home conflict.^13,20,21^ Previous literature has demonstrated that discrimination against women is linked to perceptions of a culture that is permissive of bias and harassment.^20,22,23^

Academic environments remain problematic for women.^23,24,25^ Deficits theory has been used to explain inhospitable workplaces, arguing that deficits in the scientific setting may explain differences in career experiences and outcomes because of structural mechanisms that limit opportunities for women. Examples include the so-called glass ceiling phenomenon that limits women’s abilities to advance within hierarchical organizations, the lack of universally affordable and available childcare, and restricted maternity leaves.^26^ The *organizational climate* refers to the feel of a workplace, including the shared perceptions, behaviors, and attitudes among employees.^27^ Perceptions of workplace climate may influence how employees perceive their organization and behave at work.^27^ Many organizations, such as academic centers or hospitals, may believe they provide positive, nonsexist climates; however, employees may not share these perceptions.

The Department of Medicine (DOM) at the University of Calgary Cumming School of Medicine comprises 10 sections and 389 physicians who work across multiple hospital sites in Calgary (Alberta, Canada). The purpose of this research was to explore the organizational climate for women in the DOM and further examine how physicians differ in their perceptions and experiences of gender equity.

## Methods

The study protocol was approved by the University of Calgary’s institutional ethics review board. Informed consent was obtained from all participants, including consent to publish quotations. This study follows the Consolidated Criteria for Reporting Qualitative Research (COREQ) reporting guideline^28^ and the American Association for Public Opinion Research (AAPOR) reporting definitions for survey studies.^29^

This sequential, explanatory, mixed-methods study used a quantitative strand followed by a qualitative strand, which provided further understanding and explanation of the quantitative findings from the perspective of participants.^30^ This approach generates meta-inferences by combining quantitative and qualitative data, fostering a deeper, more holistic understanding of the phenomenon of interest than either approach on its own.^30,31^ Pragmatism is the guiding framework for mixed-methods research, allowing for the practical combination and interpretation of both quantitative and qualitative data sources to generate robust inferences.

Canadian physician and medical student data are collected through self-report with only binary options for gender (ie, male or female). For this reason, we reported these statistics as female and male (referring to binary sex) rather than woman and man (referring to gender identity) as we have elsewhere in the article. In this report, we used the definitions for bias, discrimination, and harassment stated by the National Academies of Science.^23^ *Bias* refers to a set of beliefs, of which a person may be aware (explicit bias) or unaware (implicit bias), that values people differently based on characteristics, including age, sex, gender, race, ethnicity, indigenous status, and sexual orientation, which are protected by the Canadian Charter of Rights and Freedoms.^23^ *Discrimination* refers to differential actions based on these biases; these actions may be intentional or unintentional.^23^ *Harassment* refers to verbal or nonverbal actions that convey exclusion, hostility, or second-class status based on a protected characteristic.^23^

### Quantitative Strand

A cross-sectional survey was conducted using the Culture Conducive to Women’s Academic Success^22^ (CCWAS), a 45-item instrument intended to measure how conducive the workplace may be for women in academia (eMethods 1 in the Supplement). The CCWAS examines the 4 following dimensions: (1) equal access (19 items), (2) work-life balance (11 items), (3) freedom from gender biases (3 items), and (4) supportive leadership (12 items) (Table 1).^22^ All items are rated using a 5-point Likert scale ranging from 1 (ie, strongly disagree) to 5 (ie, strongly agree). Higher CCWAS scores indicate that the respondent perceives the culture to be more favorable to women. The construct of *culture* in the CCWAS refers to “the shared beliefs and expectations that contribute to the ability of women faculty to be successful in their careers.”^22^ The CCWAS has demonstrated reliability, internal consistency, and construct validity evidence.^22^ Demographic data were also collected, including gender, years in practice, faculty appointment, and parental status. The survey was distributed to all physician members in the DOM by email. The DOM email list is maintained as part of each physician member’s employment and does not contain duplicate listings. No incentive was offered for participation, and completion was voluntary.

**Table 1.** Domains and Illustrative Items of the Culture Conducive to Women’s Academic Success Instrument^a^

Domain	Items, No.	Example Item
Equal access	19	Women faculty have equal access to career development opportunities
Work-life balance	11	Colleagues are supportive when women faculty members take time for family life
Freedom from gender biases	3	Women are encouraged to raise concerns about biases against women, even if those biases are subtle
Supportive leadership	12	My chair tries to ensure that women faculty are not sexually harassed

^a^ Responses are on a 5-point Likert scale, on which 5 indicates strongly agree and 1 indicates strongly disagree.

### Qualitative Strand

We conducted 1-on-1, 1-hour semistructured interviews with physicians of any gender to further explore and explain the quantitative findings. Interviews are well-suited to discussing sensitive issues and allow participants to freely discuss their own attitudes, beliefs, perceptions, and experiences.^32^ The interviewer (G.F.) was experienced with interviewing and qualitative methods and was external to the DOM. Participation was offered through confidential email sign-up to all members of the DOM, was voluntary, and was not reimbursed. There were no exclusion criteria for participation in the interview. Purposive criterion sampling ensured that participants were faculty members or trainees affiliated with the DOM. Participants were asked to define gender equity and describe their perceptions of the current state of gender equity in the DOM. The full interview guide is provided in eMethods 2 in the Supplement.

Interviews were conducted in-person or by telephone with only the participant and interviewer present. They were audio recorded and transcribed verbatim before manual cleaning by 1 of us (G.F.) to ensure accuracy and remove identifying information before analysis. Transcripts were not provided to participants. Study team members who worked in the DOM were masked to identifying data of the participants. Participants were assigned senior or junior status using the year they graduated medical school. *Senior* was defined as having more than 15 years of practice at the time of survey distribution; these physicians were assumed to have completed medical school before 1996, when parity in the gender of medical students was achieved in Canada.^17^ Similarly, *junior* refers to physicians with 15 years or less in practice at the time of survey completion, and these participants were assumed to have graduated from medical school after 1996.

Interviews were coded using thematic analysis guided by constructivism, acknowledging the existence of multiple truths and realities.^33^ Two of us were involved in the analysis: G.F., a woman researcher external to the DOM with prior experience in qualitative methods, and S.M.R., an early-career woman physician in the DOM. Both identify as feminists. Initial codes were generated inductively through close reading of the transcripts and iteratively organized based on similarity and relationships. Codes were then reapplied to the data during a second round of coding to all transcripts. Disagreements in coding or interpretation of data were reconciled between researchers. Data were managed using Excel (Microsoft Corp).

Interviews were conducted until no additional physicians requested to participate, rather than terminating interviews based on data saturation, because DOM leadership wanted all faculty members to have the opportunity to participate. Based on the recommendations of our institutional ethics board, participants were not purposefully sampled based on characteristics such as gender, race/ethnicity, or seniority because of the sensitive nature of the study question. All interviews that were conducted were included in the analysis. There were no repeat interviews. Participants did not provide feedback on the analysis.

### Statistical Analysis

Data were analyzed using SPSS statistical software version 24 (IBM Corp). Statistical significance was set at *P* < .05, and all tests were 2-tailed. The total CCWAS score was computed for each participant. Internal consistency was evaluated using Cronbach α, and the Kaiser-Meyer-Olkin and Bartlett tests were used to examine sampling adequacy and suitability for factor analysis. A principal components analysis was conducted using promax rotation to examine the factor structure and dimensionality of the scale. The Kaiser criterion (eigenvalues, >1.0) was used to examine the number of factors, and the rotated component matrix was used to ensure all items loaded appropriately on a factor.

Nonparametric statistical tests were used to compare CCWAS scores between genders, age groups, faculty status, and parental status. Stepwise linear regression was performed to examine the association of participant characteristics with CCWAS scores. Variables included gender, years in practice, faculty status, and parental status. To examine generational differences, participants were categorized into senior and junior groups, as described earlier.

## Results

### Quantitative Results

In 2018, 171 of 389 physicians (44.0%) in DOM were women. Most women DOM members (137 [80.1%]) were younger than 50 years, compared with 112 of 218 men DOM members (51.4%). In total, 140 of 174 DOM members (80.5%) who were older than 50 years were men (eFigure in the Supplement).

The survey response rate was 43.4% (169 of 389); 102 (59.9%) were women, 65 (38.9%) were men, 2 (1.2%) preferred not to select a gender, and no participants were nonbinary (Table 2). We excluded the 2 participants who preferred not to select a gender because if any member of the DOM were openly gender diverse, their response data could be identified by reporting only these responses. Therefore, we analyzed 167 responses in total.

**Table 2.** Demographic Characteristics of Department of Medicine Survey Participants

Characteristic	No. (%)	
	Women (n = 102)	Men (n = 65)
In practice, y		
In training^a^	14 (13.7)	9 (13.9)
<2	11 (10.8)	1 (1.5)
2 to <5	13 (12.8)	6 (9.2)
5 to <10	19 (18.6)	15 (23.1)
10 to 15	17 (16.7)	8 (12.3)
>15	27 (27.0)	26 (40.0)
Prefer not to answer	1 (0.1)	0
Departmental status		
In training^a^	16 (15.7)	9 (13.8)
Major clinical, ARP	48 (47.1)	25 (38.5)
Major clinical, FFS	21 (20.6)	18 (27.7)
Geographic full time	16 (15.7)	11 (16.9)
Prefer not to answer	1 (0.1)	2 (3.1)
Parental status		
No children	27 (26.5)	15 (23.1)
Children	72 (70.6)	50 (76.9)
Prefer not to answer	3 (2.9)	0

Abbreviations: ARP, alternative relationship plan (ie, salaried academic physicians who are remunerated for clinical and nonclinical work); FFS, fee for service (ie, physicians who are primarily compensated for patients seen and procedures performed).

^a^ Two participants who selected in training for years in practice made different selections for departmental status.

The response rate among women DOM members was significantly higher than among men (excluding trainees, 88 of 171 women [51.5%] vs 56 of 218 men [25.7%]; *P* < .001). A total of 53 respondents (31.7%) had been in practice more than 15 years and were considered senior physicians. Most participants (112 [67.1%]) had a clinical faculty appointment. Overall, 122 respondents (73.1%) reported having children. There were no ineligible respondents, and there was less than 1% missingness in responses.

Cronbach α was 0.966, suggesting high internal consistency and reliability evidence for the CCWAS. The Kaiser-Meyer-Olkin statistic (0.941) and Bartlett test (*P* < .001) suggested the data were suitable for factor analysis. We extracted 7 factors with eigenvalues greater than 1, accounting for 67.9% of the total variance. All items loaded on a factor, and each factor loaded at least 2 items.

Higher CCWAS scores suggested that the respondent perceived the work culture to be more favorable for women faculty members. Women had lower median (interquartile range) CCWAS scores than men (137.0 [118.0-155.0] vs 164.5 [154.0-184.3]; *P* < .001), suggesting that men physicians perceived the culture as more favorable for women than did women members themselves. Physicians with more than 15 years in practice perceived the culture toward women as significantly more favorable than physicians with 15 years or less in practice (median [interquartile range] CCWAS, 157.0 [138.8-181.3] vs 147.0 [127.5-164.3]; *P* = .02). Residents had lower median (interquartile range) CCWAS scores than faculty members (135.0 [126.0-148.5] vs 154.0 [130.8-170.0; *P* = .01). Differences in CCWAS scores based on clinical appointment and parental status were nonsignificant (Table 3).

**Table 3.** CCWAS Scores by Group

CCWAS Score	Median (IQR)^a^	*P* Value
Gender		
Women	137.0 (118.0-155.0)	<.001^b^
Men	164.5 (154.0-184.3)	
In practice, y		
In training	135.0 (126.0-148.5)	.005^c^
<2	139.0 (115.5-151.3)	
2 to <5	152.5 (142.3-174.8)	
5 to <10	159.0 (134.5-169.3)	
10 to 15	141.0 (126.0-162.0)	
>15	157.0 (138.8-181.3)	
Departmental status		
In training	135.0 (126.0-148.5)	.09^c^
Major clinical, ARP	152.5 (135.8-170.0)	
Major clinical, FFS	152.0 (131.0-173.0)	
Geographic full time	155.0 (130.0-179.0)	
Parental status		
No children	138.0 (126.0-156.0)	.05^b^
Children	154.0 (133.8-170.0)	

Abbreviations: ARP, alternative relationship plan (ie, salaried academic physicians who are remunerated for clinical and nonclinical work); CCWAS, Culture Conducive to Women’s Academic Success; FFS, fee for service (ie, physicians who are primarily compensated for patients seen and procedures performed); IQR, interquartile range.

^a^ Higher scores indicate that the respondent perceived the culture as more favorable for women.

^b^ Calculated with Mann-Whitney test.

^c^ Calculated with Kruskal-Wallis test.

Stepwise regression found that gender and years in practice were significantly associated with CCWAS scores (*F*_2,159_ = 26.340; *P* < .001; *R*^2^ = 0.249) (Figure). Faculty and parental status remained nonsignificantly associated with CCWAS scores (faculty status: β = −0.038; *P* = .65; parental status: β = −0.105; *P* = .13).

**Figure.**
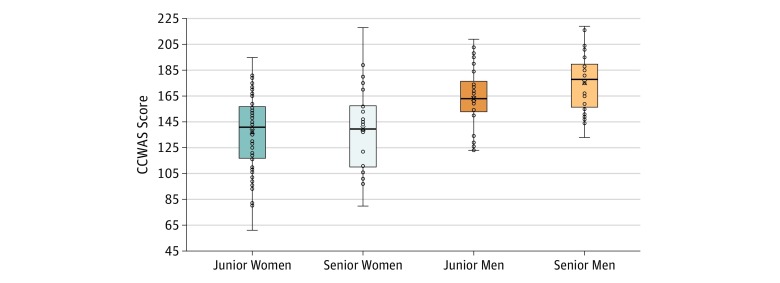
Median Scores on the Culture Conducive to Women’s Academic Success (CCWAS) by Respondents’ Gender and Years in Practice Scores on the CCWAS range from 45 to 225, with higher scores indicating that the respondent perceives the culture as more favorable to women. The horizontal lines indicate the median CCWAS score, the upper box includes the median to 75th percentile, and the lower box includes the 25th percentile to the median. The whiskers indicate the maximum and minimum CCWAS scores, and circles represent individual CCWAS responses. Junior indicates physicians with 15 or less years of work experience; senior, physicians with more than 15 years of work experience.

### Qualitative Results

Interviews were conducted with 28 physicians, representing 7.2% of the DOM. Overall, 20 junior women (71.4%), 2 senior women (7.1%), 2 junior men (7.1%), and 4 senior men (14.2%) participated in interviews, representing 12.9% of the women (22 of 171) and 2.8% of the men (6 of 218) in the DOM (Table 4).

**Table 4.** Department of Medicine Interview Participants by Gender and Age

Status	No. (%)^a^				*P* Value
	Men (n = 218)		Women (n = 171)		
	Participants	Nonparticipants	Participants	Nonparticipants	
Senior, >15 y in practice	4 (3.7)	102 (96.2)	2 (5.9)	32 (94.1)	NA
Junior, ≤15 y in practice	2 (1.8)	110 (98.2)	22 (16.1)	115 (83.9)	NA
Total	6 (2.8)	212 (97.2)	24 (14.0)	147 (86.0)	<.001

^a^ Response rate is calculated for each demographic within the Department of Medicine.

We derived 20 codes during the initial round of coding, and 3 additional codes were added on the second round; 7 codes were associated with interventions proposed by the study team and are not reported in this article. Multiple levels were allowed for certain codes. The final codebook appears in eTable 1 in the Supplement.

Interviewees were asked to define gender equity, their perceptions of gender equity in the DOM, and their exposure to gender inequities within the department, either as first-hand experiences or observed in the lives and careers of women colleagues. Exposure to gender inequity fell into the 4 following thematic areas: (1) harassment and discrimination, (2) parenthood and caregiving, (3) exclusion (ie, lack of access to leaders, mentors, or opportunities) and (4) career growth and opportunities. Subthemes often overlapped; for example, participants often related exclusion from leadership opportunities to maternal discrimination. We examined the major themes from the perspectives of junior women, junior men, senior women, and senior men physicians. Supportive quotes for each theme appear in eTable 2 in the Supplement.

#### Definitions of Gender Equity

All demographic groups defined gender equity as equal access to opportunity and the absence of bias. Men faculty explicitly stated that numerical equality of men and women in a committee, leadership roles, or employment was not necessary for or synonymous with gender equity. Junior women participants emphasized how innate differences between men and women could lead to differential barriers to success and defined gender equity as unequal action to reduce these barriers. For example, participant 3, a junior woman physician, stated, “We’re not exactly the same, and so it’s equity within our differences.... Women do take breaks to have children, and so what are the supports... to make sure that their trajectories are in line with men?”

#### Perceptions of Gender Equity at the DOM

Senior men participants predominantly expressed that there was no gender inequity in the DOM in its current state; many provided examples of numerical parity as evidence that gender equity had been achieved in the DOM. For some respondents, these statements contradicted their definition of gender equity as being different than numerical equality. A junior woman physician (participant 8) provided an example of men DOM members using numerical parity as an example of gender equity: “I am sitting on a committee right now.... [The man leader] pointed out there was gender equity in terms of who was sitting around the table, but you look around... [and] 3 out of the 6 or 7 women... were in secretarial or support roles..., whereas all of the men around the table were... all going to be voting members.”

Junior men, junior women, and senior women reported that gender equity in the DOM was “improving” but noted that there was still “room for improvement” (participant 13, a junior woman). Most women participants considered their experiences with gender equity at the DOM to be generally good, but all recalled specific instances where they had experienced gender bias in their professional lives. Three men participants acknowledged that, as men, they may be less aware of gender-based discrimination in the DOM. Of these 3 participants, 2 noted that recent conversations (around the experience of underrepresented groups in medicine) and personal experiences (becoming a parent) had increased their awareness of possible gender inequities within the DOM. For example, participant 12, a junior man physician said, “Since we had my son… I’m a bit more sensitive to issues relating to children, relating to gender equity.”

#### Experiences of Gender Inequity

##### Harassment and Discrimination

Women physicians described explicit harassment and discrimination in their professional lives (harassment and discrimination were self-defined by participants or were retrospectively defined by coders based on agreed-on definitions^23^). Junior men participants described witnessing discrimination experienced by women colleagues. In contrast, senior men participants did not report any instances of observing or experiencing harassment or discrimination within the DOM.

Women participants distinguished this deliberate harassment and discrimination from unintentional biases and frequently attributed unintentional biases to lack of insight by their men colleagues; the lack of insight included both the consequences of their words and actions on women physicians and a lack of awareness of the unique challenges that women physicians experience. For example, participant 1, a junior woman physician, said, “[Our men colleagues] are inherently impaired from being able to see some of the biases that exist.… When you’re part of the majority sometimes it’s hard to see the challenges that people that aren’t a part of your social group face.”

##### Parenthood and Caregiving

Junior women participants highlighted a lack of awareness among men colleagues of their additional responsibility in caregiving and home responsibilities as physician-mothers. Multiple participants felt that this lack of understanding facing physician-mothers represented a significant barrier to effective mentorship, led to incomplete assessments of their dedication to work, and resulted in frank discrimination. Participant 8 said, “[My male mentor] said ‘That’s what Saturday afternoons are for, that’s when you do your research,’ and I just... said, ‘That’s when I do my laundry.’... He stopped to think.... He has a partner at home who his entire career and life has done the laundry, the life maintenance at home.”

Three men physicians acknowledged having an incomplete understanding of the challenges faced by physician-mothers and questioned whether there were adequate supports available from the DOM. Both junior men physicians expressed an interest in maintaining a work-life balance that allowed for career advancement and active participation in family life but were uncertain of how this balance would be met in the DOM. For example, participant 5 stated, “Would my ability to [be] productive, accomplish as much as I hope be the same …in a family scenario? [I] certainly have had that fear, anticipation of that possibility.”

##### Exclusion

Men participants did not note the existence of gender-based social exclusion; however, they did acknowledge that access to mentors was key to career advancement and opportunities. Women reported experiencing social exclusion that isolated them from colleagues, division and department leaders, potential mentors, and career opportunities. Participant 1, a junior woman physician, said, “My leaders in medicine [will suggest] we go to dinner and drinks.... As a woman, I don’t really like having drinks; that’s not a space I want to actually have a professional meeting at, and I’ve got 3 kids to feed..., [but] if you’re not available for [these events] then you’re going to be disadvantaged.”

##### Career Growth and Opportunities

Women participants perceived disparity in career progression between men and women colleagues. Multiple women participants cited specific examples of men colleagues who were initially hired in more senior academic positions with perceived equal qualifications to the participant, and many women participants reported feeling similarly bypassed for leadership roles. Men did not perceive disparities in hiring or career progression but did note a lack of clarity around selection of leadership roles. Several junior women reported that their awareness of subtle and unintentional biases has increased throughout their career, such that they felt that the effects of gender were much less in medical training and residency but had a greater effect as they became staff. Junior women emphasized that while many of their experiences of discrimination were subtle and difficult to prove as gender related, they were numerous, consistent, and affected well-being, career satisfaction, and career trajectory. For example, participant 19 stated, “I look at my male colleagues who are very confident and very accomplished and doing great things… because they’ve been given the support to achieve whatever it is they want to achieve.... How great it would be to see females be able to do that with complete support to achieve [what] they want. Instead I see women… giving up the fight because it’s too exhausting.”

#### Generational Explanations for Gender Inequity in Medicine

Junior women participants openly discussed generational differences in beliefs about and experiences of gender inequity in medicine. Junior women were aware that senior men colleagues did not believe that gender inequities existed but did not feel comfortable contradicting senior men physicians who had been vocal in stating that there was no bias in the DOM. Men noted a generational shift in the culture of medicine both in the pursuit of gender equity as a goal and in the increased interest in paternal leave and work-life balance.

Both junior women and men physicians felt that the solution to these generational challenges was to “wait until the old guard die or retire” because older men physicians were “afraid of change,” as participant 24, a junior woman participant, said. Several junior women respondents reported discrimination or harassment from junior men colleagues, which they attributed to role-modeling, acceptance of these behaviors by leadership, and an overall culture in medicine that devalues women.

## Discussion

This sequential, explanatory, mixed-methods study of experiences of gender equity in the DOM at the University of Calgary found different perceptions of the culture toward women in the DOM between men and women physicians and, additionally, between junior and senior physicians. Senior men and junior women physicians’ views on gender equity in medicine were the most divergent.

In our study, senior men perceived gender equity to be achieved in the DOM and used numerical equality as evidence of lack of bias against women physicians. Senior men did not recognize barriers to a career in medicine that women physicians might face, such as parenthood, second-shift responsibilities, and sexual harassment. In contrast, junior men, junior women, and senior women physicians perceived gender inequities in medicine that disadvantaged women physicians. These 3 groups had increased awareness of the range of challenges faced by women physicians and the consequences of these challenges on career progression. Further, junior women and men physicians shared concerns about the effect of parenthood on career progression.

Senior men physicians were trained in a medical culture with less exposure to the challenges faced by early-career women physicians. Physicians who are older than 50 years likely graduated from medical school before 1996, when the gender distribution of medical school was different. In 1996, only 27% of practicing physicians and 48% of medical students in Canada were women.^17^ Earlier graduating classes had even fewer female medical students. In this environment, there may have been less open discussion of implicit or subtle biases by their women colleagues, contributing to less recognition by men physicians of the burden and consequences of these biases. These cultural and demographic factors may have contributed to a lack of awareness by senior men physicians of barriers faced by women physicians.

In contrast, early-career and midcareer men physicians trained with near parity of women. The increased exposure to barriers faced by their peers may help to explain why junior men physicians’ views on gender equity align with those of junior women. In keeping with this, junior men participants were able to describe examples of implicit and explicit discrimination toward their women peers and had greater recognition of barriers faced, such as family responsibilities, second-shift duties, and sexual harassment. Junior men also demonstrated an understanding of the effect of these experiences on their women colleagues’ careers and wellness.

Previous literature has demonstrated generational and gender-based differences in experiences and values in medical careers. In general, recent medical graduates of any gender may be less likely to prioritize work as a physician over time with their families than older men physicians; data show that older men physicians work more hours per week than younger men and women physicians.^34,35,36^ Younger men physicians are more likely than older men physicians to reduce work hours to accommodate children and marriage and are more likely to be supportive of their female colleagues during pregnancy, maternity leave, and early parenthood.^37,38,39^ In addition, younger men and women physicians are more likely to take parental leave than previous generations of physicians.^40^ Further, contemporary women physicians are less likely than their physician mothers to feel that caregiving has slowed their career trajectories.^40^

Generational and gender differences in values for physicians have important consequences for junior physicians. In 2018, 42% of physicians and 57% of medical students in Canada were female; however, 70% of physicians older than 55 years were male compared with 61% of physicians younger than 55 years (eFigure in the Supplement).^17^ This is reflected in Canadian medical leadership; currently, 88% of medical school deans and 69% of the Canadian Medical Association executive team is composed of men.^15,16^ Women physicians are concentrated in younger age categories, where many of the barriers, such as salary negotiation at career entry, pregnancy and childbearing, and gender-based and sexual harassment, are experienced; however, decisions and policies on antiharassment strategies, parental leave, breastfeeding accommodations, and promotion are predominantly made by men physicians. A directed study examining the perceptions and values related to equity, diversity, and inclusion of men physician decision-makers and the impact of these values on policy making that affects women physicians is an important next step.

### Limitations

The present study is limited in that it represents participants at a single department, therefore representing our specific institutional culture and limiting the generalizability of our data. We did not actively recruit participants from specific demographics owing to ethical issues and had low numbers of senior women and junior men interview participants. This limited our ability to draw conclusions about the perceptions and values of these groups and is an area of future study. All interview participants identified as a man or woman, and the survey did not collect data on race or ethnicity; both gender nonconformity and racialized individuals are expected to have different experiences of harassment, equity, and inclusion and are even more underrepresented in leadership positions. All participants were self-referred and therefore response bias is expected. In particular, individuals with stronger opinions or experiences may be more likely to participate, and therefore, examples of harassment, discrimination, and exclusion may be overrepresented in our sample compared with the study population.

## Conclusions

This mixed-methods study suggested a demographic mismatch characterized by differences in perceptions and experiences of gender equity between men and women physicians and between senior and junior physicians in an academic center in Canada. Senior men differed most from junior women in perceptions of the climate for women physicians. This is important as senior men physicians hold most decision-making and leadership positions in Canada, but these decisions will affect junior women physicians. This gap between perceptions of decision-makers and experiences of end-users may explain the failure of women physicians to progress in medicine, despite 25 years of gender parity in Canadian medical graduates.

## Notes

Supplement. **eMethods 1. **Survey Instrument **eMethods 2. **Gender Equity Interview Guide **eFigure. **Population Pyramid of Department of Medicine Physician Members and Canadian Physicians in 2018 **eTable 1. **Final Coding Framework for Interviews **eTable 2. **Exemplary Quotes for Each Participant Demographic and Major Theme Identified From the Qualitative Strand
