# Supplementary material for: Association of Physician Characteristics With Perceptions and Experiences of Gender Equity in an Academic Internal Medicine Department
Source: JAMA Netw Open. 2019 Nov 13;2(11):e1915165. doi: 10.1001/jamanetworkopen.2019.15165 (PMC6902791; doi:10.1001/jamanetworkopen.2019.15165)
Supplement: Supplement. — eMethods 1. Survey Instrument eMethods 2. Gender Equity Interview Guide eFigure. Population Pyramid of Department of Medicine Physician Members and Canadian Physicians in 2018 eTable 1. Final Coding Framework for Interviews eTable 2. Exemplary Quotes for Each Participant Demographic and Major Theme Identified From the Qualitative Strand [file jamanetwopen-2-e1915165-s001.pdf]

Supplementary Online Content

Ruzycki SM, Freeman G, Bharwani A, Brown A. Association of physician characteristics with perceptions and experiences of gender equity in an academic internal medicine department. *JAMA Netw Open*. 2019;2(11):e1915165.
doi:10.1001/jamanetworkopen.2019.15165

eMethods 1. Survey Instrument
eMethods 2. Gender Equity Interview Guide
eFigure. Population Pyramid of Department of Medicine Physician Members and Canadian Physicians in 2018
eTable 1. Final Coding Framework for Interviews
eTable 2. Exemplary Quotes for Each Participant Demographic and Major Theme Identified From the Qualitative Strand

This supplementary material has been provided by the authors to give readers additional information about their work.

© 2019 Ruzycki SM et al. *JAMA Network Open.*

eMethods 1. Survey Instrument

Instructions

Your responses to the survey are anonymous. There are 60 questions and the survey is estimated to take about 10 minutes total. There is a comment box at the end of the survey for all comments, concerns, and additional information. Thank you for your time.

1. What is your gender?
   - o Male
   - o Female
   - o Other (please specify)

2. How many years have you been in clinical practice?
   - o Currently in training
   - o < 2 years
   - o 2-5 years
   - o 5-10 years
   - o > 15 years
3. Which of the following best describes your role in the Department of Medicine?
   - o In training
   - o GFT
   - o Major Clinical
   - o Fee for Service
   - o Major Clinical
   - o ARP

4. Do you have children?
   - o Yes
   - o No
   - o Prefer not to answer

5. In general, in my department, compared to men faculty, women faculty have equal access to career development opportunities.
   - o Strongly agree
   - o Agree
   - o Neither agree not disagree
   - o Disagree Strongly
   - o Disagree

6. In general, in my department, compared to men faculty, women faculty get as much mentoring from senior faculty.
   - o Strongly agree
   - o Agree
   - o Neither agree not disagree
   - o Disagree Strongly
   - o Disagree

7. In general, in my department, compared to men faculty, women faculty are as frequently considered for leadership positions.
   - o Strongly agree
   - o Agree
   - o Neither agree not disagree

- o Disagree Strongly
- o Disagree

8. In general, in my department, compared to men faculty, women faculty receive as much feedback regarding their performance.
    - o Strongly agree
    - o Agree
    - o Neither agree not disagree
    - o Disagree Strongly
    - o Disagree
9. In general, in my department, compared to men faculty, women faculty receive as much guidance about potential research opportunities.
    - o Strongly agree
    - o Agree
    - o Neither agree not disagree
    - o Disagree Strongly
    - o Disagree

10. In general, in my department, compared to men faculty, women faculty receive equitable salaries.
    - o Strongly agree
    - o Agree
    - o Neither agree not disagree
    - o Disagree Strongly
    - o Disagree

11. In general, in my department, compared to men faculty, women faculty get as much research space/equipment.
    - o Strongly agree
    - o Agree
    - o Neither agree not disagree
    - o Disagree Strongly
    - o Disagree

12. In general, in my department, compared to men faculty, women faculty get as much office space.
    - o Strongly agree
    - o Agree
    - o Neither agree not disagree
    - o Disagree Strongly
    - o Disagree

13. In general, in my department, compared to men faculty, women faculty have equal access to administrative support.
    - o Strongly agree
    - o Agree
    - o Neither agree not disagree
    - o Disagree Strongly
    - o Disagree

14. In general, in my department, compared to men faculty, women faculty have LESS protected time for research.
    - o Strongly agree
    - o Agree
    - o Neither agree not disagree
    - o Disagree Strongly
    - o Disagree

15. In general, in my department, compared to men faculty, women faculty are as frequently recognised for their work.

© 2019 Ruzycki SM et al. *JAMA Network Open.*

- Strongly agree
- Agree
- Neither agree not disagree
- Disagree Strongly
- Disagree

16. In general, in my department, compared to men faculty, women faculty are as often asked to sit on prestigious committees.
    - Strongly agree
    - Agree
    - Neither agree not disagree
    - Disagree Strongly
    - Disagree

17. In general, in my department, compared to men faculty, women faculty are as frequent nominated for awards and honuors.
    - Strongly agree
    - Agree
    - Neither agree not disagree
    - Disagree Strongly
    - Disagree

18. In general, in my department, compared to men faculty, women faculty are more likely to have others take credit for their work.
    - Strongly agree
    - Agree
    - Neither agree not disagree
    - Disagree Strongly
    - Disagree

19. In general, in my department, compared to men faculty, women faculty are as frequently included in discussion of division policies and administration.
    - Strongly agree
    - Agree
    - Neither agree not disagree
    - Disagree Strongly
    - Disagree

20. In general, in my department, compared to men faculty, women faculty play equally important roles in decision-making.
    - Strongly agree
    - Agree
    - Neither agree not disagree
    - Disagree Strongly
    - Disagree

21. In general, in my department, compared to men faculty, the comments made by women faculty in meetings are given as much credit and attention.
    - Strongly agree
    - Agree
    - Neither agree not disagree
    - Disagree Strongly
    - Disagree

22. In general, in my department, compared to men faculty, women faculty are as frequently included in professional social gathers (e.g. dinners with guest scientists)

© 2019 Ruzycki SM et al. *JAMA Network Open.*

- Strongly agree
- Agree
- Neither agree not disagree
- Disagree Strongly
- Disagree

23. In general, in my department, colleagues are supportive when women faculty members talk about work-family issues.
    - Strongly agree
    - Agree
    - Neither agree not disagree
    - Disagree Strongly
    - Disagree

24. In general, in my department, attending to personal needs, such as taking time off for sick children, is frowned upon.
    - Strongly agree
    - Agree
    - Neither agree not disagree
    - Disagree Strongly
    - Disagree

25. In general, in my department, women faculty who reduce their workload are viewed by their colleagues as less committed to their careers.
    - Strongly agree
    - Agree
    - Neither agree not disagree
    - Disagree Strongly
    - Disagree

26. In general, in my department, family demands are considered when the division schedules events and/or meetings.
    - Strongly agree
    - Agree
    - Neither agree not disagree
    - Disagree Strongly
    - Disagree

27. In general, in my department, family demands are considered when the division schedules teaching and clinical hours.
    - Strongly agree
    - Agree
    - Neither agree not disagree
    - Disagree Strongly
    - Disagree

28. In general, in my department, an obstacle for full-time women faculty is the expectation of a minimum of a 60-hour work week.
    - Strongly agree
    - Agree
    - Neither agree not disagree
    - Disagree Strongly
    - Disagree

29. In general, in my department, reducing their work load hurts the chances that women faculty will succeed in their careers.

© 2019 Ruzycki SM et al. *JAMA Network Open.*

- Strongly agree
- Agree
- Neither agree not disagree
- Disagree Strongly
- Disagree

30. In general, in my department, women faculty who temporarily reduce their work load or parenting responsibilities are expected to take on extra work when they return to full time.
    - Strongly agree
    - Agree
    - Neither agree not disagree
    - Disagree Strongly
    - Disagree

31. In general, in my department, work is expected to be the primary focus of faculty members' lives.
    - Strongly agree
    - Agree
    - Neither agree not disagree
    - Disagree Strongly
    - Disagree

32. In general, in my department, it is possible for women faculty to get promoted working 50 hours per week or less on a regular basis.
    - Strongly agree
    - Agree
    - Neither agree not disagree
    - Disagree Strongly
    - Disagree

33. In general, in my department, colleagues are supportive when men faculty members take time for family life.
    - Strongly agree
    - Agree
    - Neither agree not disagree
    - Disagree Strongly
    - Disagree

34. In general, in my department, colleagues are supportive when men faculty members talk about work-family issues.
    - Strongly agree
    - Agree
    - Neither agree not disagree
    - Disagree Strongly
    - Disagree

35. In general, in my department, male faculty who reduce their workload are viewed by their colleagues as less committed to their careers.
    - Strongly agree
    - Agree
    - Neither agree not disagree
    - Disagree Strongly
    - Disagree

36. In general, in my department, an obstacle for full time male faculty is the expectation of a minimum of a 60-hour work week.
    - Strongly agree
    - Agree

- Neither agree not disagree
- Disagree Strongly
- Disagree

37. In general, in my department, reducing their work load hurts the chances that male faculty will succeed in their careers.
    - Strongly agree
    - Agree
    - Neither agree not disagree
    - Disagree Strongly
    - Disagree

38. In general, in my department, male faculty who temporarily reduce their work load for parenting responsibilities are expected to take on extra work when they return to full time.
    - Strongly agree
    - Agree
    - Neither agree not disagree
    - Disagree Strongly
    - Disagree

39. In general, in my department, it is possible for male faculty to get promoted working 50 hours per week or less on a regular basis.
    - Strongly agree
    - Agree
    - Neither agree not disagree
    - Disagree Strongly
    - Disagree

40. In general, in my department, women faculty members are comfortable raising issues about the supportiveness of the work environment for women.
    - Strongly agree
    - Agree
    - Neither agree not disagree
    - Disagree Strongly
    - Disagree

41. In general, in my department, women are encouraged to raise concerns about biases against women, even if those biases are subtle.
    - Strongly agree
    - Agree
    - Neither agree not disagree
    - Disagree Strongly
    - Disagree

42. In general, in my department, when women faculty raise concerns about gender issues, they are seen as “whiners”.
    - Strongly agree
    - Agree
    - Neither agree not disagree
    - Disagree Strongly
    - Disagree

43. In general, in my department, my division head tries to ensure that women faculty have equal access to support and resources (e.g. space, administrative support, career development opportunities) to help them in their careers compared to men faculty.
    - Strongly agree

© 2019 Ruzycki SM et al. *JAMA Network Open.*

- Agree
- Neither agree not disagree
- Disagree Strongly
- Disagree

44. In general, in my department, my division head tries to ensure that women faculty are equally recognised and rewarded for their work compared to men faculty.
    - strongly agree
    - Agree
    - Neither agree not disagree
    - Disagree Strongly
    - Disagree

45. In general, in my department, my division head tries to ensure that women faculty are included in FORMAL division events.
    - Strongly agree
    - Agree
    - Neither agree not disagree
    - Disagree Strongly
    - Disagree

46. In general, in my department, my division head tries to ensure that women faculty are included in INFORMAL division gatherings (e.g. coffee, lunches, sporting events, etc.)
    - Strongly agree
    - Agree
    - Neither agree not disagree
    - Disagree Strongly
    - Disagree

47. In general, in my department, my division head is supportive when women faculty talk about work-family issues.
    - strongly agree
    - Agree
    - Neither agree not disagree
    - Disagree Strongly
    - Disagree

48. In general, in my department, my division head encourages women faculty to take advantage of policies/practices for managing work and family.
    - Strongly agree
    - Agree
    - Neither agree not disagree
    - Disagree Strongly
    - Disagree

49. In general, in my department, my division head ensures work coverage for women faculty on maternity leave.
    - Strongly agree
    - Agree
    - Neither agree not disagree
    - Disagree Strongly
    - Disagree

50. In general, in my department, my division head sends a message that parenthood is an expected part of life.
    - Strongly agree
    - Agree
    - Neither agree not disagree

© 2019 Ruzycki SM et al. *JAMA Network Open.*

- Disagree Strongly
- Disagree

51. In general, in my department, my division head tries to ensure that women faculty are able to manage the demands of work and family.
    - Strongly agree
    - Agree
    - Neither agree not disagree
    - Disagree Strongly
    - Disagree

52. In general, in my department, my division head tries to ensure that women faculty feel free to express concerns regarding the treatment of women.
    - Strongly agree
    - Agree
    - Neither agree not disagree
    - Disagree Strongly
    - Disagree

53. In general, in my department, my division head tries to ensure that women faculty are not sexually harassed.
    - Strongly agree
    - Agree
    - Neither agree not disagree
    - Disagree Strongly
    - Disagree

54. In general, in my department, my division head tries to ensure that women faculty are not subject to subtle gender-based biases.
    - Strongly agree
    - Agree
    - Neither agree not disagree
    - Disagree Strongly
    - Disagree
55. Are there any programs or interventions that you would like to see the Department of Medicine trial to improve gender equity.

56. Please use this space to leave any information you want the study team to know about gender equity. This may include barriers to gender equity that you have experienced, anecdotes, stories, comments and/or concerns. These comments are anonymous.

eMethods 2. Gender Equity Interview Guide

Introduction

The purpose of the interview is to bear witness to gender equity in the Department of Medicine (DOM) and to gather feedback on six proposed interventions designed by the Gender Equity Task Force intended to reduce gender inequities.

Interview participants have completed an online survey adapted from the Culture Conducive to Women in Academic Medicine survey (Appendix I), an instrument validated in female and male physicians working in academia to assess workplace gender culture. Interview participants have self-identified themselves as interested in undergoing an in-person interview. Some participants have been recruited by the study team.

Part One: Consent and Information for Further Support (5 minutes)

Please provide each participant with the Interview Consent form and answer all questions related to the consent (Appendix II). Please ensure that all participants sign the consent form.

Please highlight the following information in the section "What are the risks":

> *The purpose of the interview is to assess gender equity in the Department of Medicine. Some of the questions are of a sensitive nature and may be upsetting. If you are struggling with issues related to gender inequity or harassment, please contact the Physician Family Support Program (1-877-767-4637) or the University of Calgary sexual harassment advisor, Shirley Wilson (403-220-4086 or wsvoyna@ucalgary.ca).*

Part Two: Bearing Witness/Assessing Barriers to Gender Equity (20 minutes)

We would like to follow-up on questions in the pre-interview survey. The participant's responses will not be available as the survey was anonymous. Each participant will be provided with a hard copy of the survey.

Please ask each participant which questions they would like to discuss and elaborate on. Please ask for specific examples or supporting details as needed.

Part Three: Identifying Potential Facilitators of Gender Equity (25 minutes)

Please ask each participant:

1. What does gender equity mean to you?

   (*Follow-up probe questions include: Is there gender equity in the Department of Medicine currently? What has been your experience with gender equity in the Department of Medicine?)*

2. Have you experienced barriers to progress in your career? What did those barriers look like? (*Prompt: some potential challenges you might have experienced could be with work-life balance, systemic issues or barriers, or a need for mentorship*)

3. What programs or policies could the DOM implement that would improve your experience with gender equity in the department? (*Please gather details as appropriate*)

© 2019 Ruzycki SM et al. *JAMA Network Open.*

eFigure. Population Pyramid of Department of Medicine Physician Members and Canadian Physicians in 2018

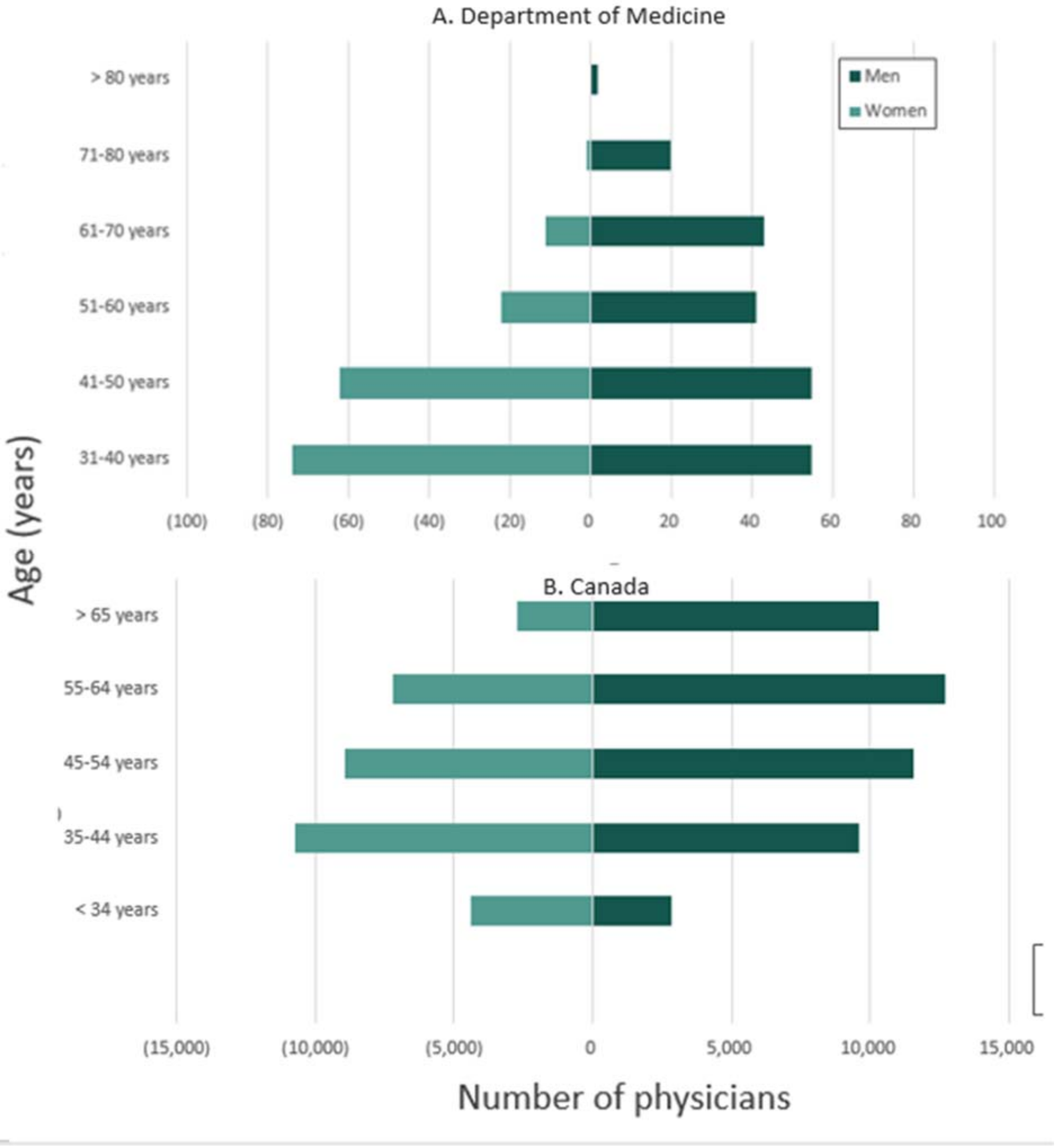

© 2019 Ruzycki SM et al. *JAMA Network Open.*

eTable 1. Final Coding Framework for Interviews

Code	Levels	Description
1		Definition of gender equity
2		Measurement and evidence for gender equity – explicit mentions of presence, absence or need for evidence (distinct from anecdotal statements of experience)
3		Recommendations or suggested interventions to address gender disparities.
4	A – Career entry<br>B – Early career (defined by participant)<br>C – Career progression or promotion	Career
5	A – Access to opportunities	Opportunities - committee membership, etc
6		Awards and recognition(acknowledgement or credit for work/achievement)
7		Grants
8		Finances
9		Mentorship - includes seeking advice/support
10		Value metrics - including values without a metric
11	A – Anticipation, planning, decision-making, actions taken<br>B – Parental leave	Parenthood
12	A – Experience of equity	Experience of bias (non-department, non-divisional)
13	A – perception of gender equity in leadership<br>B – Opportunities for leadership	Leadership (formal, committee leads)
14	A – Specific mention of Division Head<br>B – Division culture<br>C – Division-specific experience of bias	Division specific-experience
15	A – Specific mention of Department Head<br>B – Department culture<br>C – Department-specific experience of bias	Department specific experience
16		Social Events
	*	Added to code when the participant is relaying a general perception of what is happening in the Department
	+	Added to code when the participant is relaying a story that happened to a friend or colleague

© 2019 Ruzycki SM et al. *JAMA Network Open.*

eTable 2. Exemplary Quotes for Each Participant Demographic and Major Theme Identified From the Qualitative Strand

“Junior” refers to participants who graduated medical school after 1996 and “Senior” refers to participants who graduated before 1996. Participant gender was self-identified.

Themes		Exemplar Quotes from Each Represented Demographic			
		Junior Females (n=20)	Junior Males (n=2)	Senior Females (n=2)	Senior Males (n=4)
Gender equity is…	…absence of bias.	“being treated fairly with respect in value, regardless of if you’re male or female, white, black” (P19)	“I would hope that gender equity would ...include that blindness to a person’s gender and their ability to contribute” (P05)	“[Gender equity is when] gender isn’t a barrier to advancement” (P15)	“hire the best mind that you can find, bet it female, black or white doesn’t matter, you hire the mind not the skin” (P06)
	…equal opportunity.	“I think it would mean that females and males would have the same opportunities … that they can advance in their careers and be as supported and as successful as males” (P04)	“equal opportunities for every gender whether that be opportunities to pursue their clinical, educational, academic, research agenda” (P12)	“equal opportunity, equal respect regardless of your gender” (P22)	“equal opportunities … for happiness, for contentedness, for satisfaction in life’s choices and career choices and personal choices” (P25)
	…non-numerical.		“I don’t think you need to be 50/50 … the best person should get the job, …if that results in an 80% female leadership then that’s great, and if it’s a 30% female leadership, then I mean if they’re the best people and if the best person is getting the done, maybe that’s the best thing” (P05)		“not necessarily that you have to be numerically 50% of anything, it doesn’t have to be 50/50 on every front because I think that’s a silly quota, irrespective of which gender is more represented” (P14)
	…respect for differences.	“equity sometimes means that people need extra support to get to the same spot” (P20)			

© 2019 Ruzycki SM et al. *JAMA Network Open.*

Themes		Sample Quotes from Each Represented Demographic			
		Junior Females (n=20)	Junior Males (n=2)	Senior Females (n=2)	Senior Males (n=4)
Gender equity at the DOM is…	…achieved.				“I would say that this Department of Medicine is doing stalwart work at achieving gender equity …at my hospital we have a you know coincidentally a 50/50 split in terms of the physicians who are present at our hospital” (P14)
	… improving but imperfect	"As a young female physician, I feel like a very valued member with tons of support throughout the department that is totally gender neutral... but it's those snippets that catch you off guard and you're like 'Ok there is something else going on here'" (P03)	“We’re working towards gender equity because we’re not there yet, and I mean I’d like to think …our generation is a lot more aware and a lot better at it than previous generations but I think there’s still a long ways to go” (P12)	"Within the DOM, everybody knows each other, and we support each other... [but] there are certain individuals who you know you can never rely on to help" (P15)	
	…uncertain.		“In terms of you know other concrete things the department has done or that I’ve been exposed to I haven’t seen specific gender biases myself, but that might be limited as a male participant in this process as opposed to a female participant right” (P05)		“I believe we’re doing pretty good but I say that as a male unconscious of data that …might substantiate whether we’re actually doing that or not” (P25)

© 2019 Ruzycki SM et al. *JAMA Network Open.*

Themes		Exemplar Quotes from Each Represented Demographic			
		Junior Females (n=20)	Junior Males (n=2)	Senior Females (n=2)	Senior Males (n=4)
Experiences of bias related to:	Harassment and discrimination	“Another male specialist in my division said 'I'm fine if [women] want to try and do this job, but for god's sake, please buy a bit of lipstick or put on a dress'” (P19)	“I have …borne witness to what I think is disparity between genders … I’ve seen [patients and allied health professionals] talk to me as opposed to my female [superior] who is standing beside me“ (P05)	"I've been to division meetings where crazy stuff is said, so sexist, and [our division head] doesn't ... say anything... most men have very poor insight that they're being sexist"(P22)	
	Parenthood and caregiving	“I had a male colleague come to me and say… ‘we have had all these meetings, why couldn’t you come… it shows you don’t care about your career’ … you know how hard it is to get to these meetings at seven in the morning [with] kids at that [young] age” (P13)	“There are barriers for women in particular who take …mat leave or take time off to have children… I think being able to come back to your role and still maintain the same trajectory is the most important thing and I wonder whether we’re making adequate steps …to do that” (P12)	“the comment from two men was well she’s no doctor [name redacted]… and I was the one who had to point out .. she has … young children … how does that fit into your perspective of where people should be at this stage in their career” (P22)	“most of the women are the family caregivers as well and there probably are issues around duty hours that I don’t even understand, that have to do with you know family responsibilities and family duties” (P14)
	Exclusion (lack of access to leaders, mentors and opportunities)	“There’s a lot of mentorship that happens behind the scenes … if you’re not even being invited to the golf course … you just lost potentially a massive opportunity for some mentorship or career advancement” (P08)	“I would hate to think the department is missing out on the best person because we’re not pushing or mentoring our female colleagues just as much as our male colleagues.” (P05)	“You attend conferences together, … but again you’re not part of the group … you make no connections here, I mean it’s a wonder I got a job here” (P22)	“I think that it would be really valuable to have senior men leaders prepared to be mentors to junior female colleagues … so that they can you know have an equitable access to leadership opportunities, advancement“ (P14)
	Career growth and opportunities	"They seem to be more willing to put males into clinical assistant professor roles right off the bat before making them prove themselves... a lot of the new faculty members, the males, are getting hired into higher roles even if they haven't necessarily done a ton of work whereas the females are not" (P04)	“I’d say the biggest [barrier to career progression] is … ambiguity in some of the process of being hired and being promoted” (P05)	“I’ve heard the stories from other women about you know being, feeling like they were bypassed or letting the person they report to know they were very interested in a position just to find out that the position wasn’t even posted and given to somebody else, which to me is just horrible leadership” (P15)	“I suppose sometimes there’s an assumption that people know what they need to do in order to move up through the ranks and that’s not always the case” (P02)

© 2019 Ruzycki SM et al. *JAMA Network Open.*

Themes	Sample Quotes from Each Represented Demographic			
	Junior Females (n=20)	Junior Males (n=2)	Senior Females (n=2)	Senior Males (n=4)
Generational differences in perceptions of inequity	"The older, bigger component of my [division] is... very strong voices, male voices that are a little bit against women, definitely they don't see us as equal or as well trained or as dedicated, they see us [as] different" (P16)	"Our division is primarily a male division, so I think in the past ... [taking parental leave] wasn't something that had to be entertained so [it] was ... a new situation for our division to go through" (P12, referring to taking a paternity leave)		"It's not the usual culture of medicine, senior physicians generally not accustomed to ... getting much in the way of feedback so [pursuing gender equity] would be a culture change" (P14)

© 2019 Ruzycki SM et al. *JAMA Network Open.*
